# Transferring the Characteristics of Naturally Occurring and Biased Antibody Repertoires to Human Antibody Libraries by Trapping CDRH3 Sequences

**DOI:** 10.1371/journal.pone.0043471

**Published:** 2012-08-24

**Authors:** Sophie Venet, Ulla Ravn, Vanessa Buatois, Franck Gueneau, Sébastien Calloud, Marie Kosco-Vilbois, Nicolas Fischer

**Affiliations:** NovImmune SA, Geneva, Switzerland; Center for Genomic Regulation, Spain

## Abstract

Antibody repertoires are characterized by diversity as they vary not only amongst individuals and post antigen exposure but also differ significantly between vertebrate species. Such plasticity can be exploited to generate human antibody libraries featuring hallmarks of these diverse repertoires. In this study, the focus was to capture CDRH3 sequences, as this region generally accounts for most of the interaction energy with antigen. Sequences from human as well as non-human sources were successfully integrated into human antibody libraries. Next generation sequencing of these libraries proved that the CDRH3 lengths and amino acid composition corresponded to the species of origin. Specific CDRH3 sequences, biased towards the recognition of a model antigen either by immunizing mice or by selecting with phage display, were then integrated into another set of libraries. From these antigen biased libraries, highly potent antibodies were more frequently isolated, indicating that the characteristics of an immune repertoire is transferrable via CDRH3 sequences into a human antibody library. Taken together, these data demonstrate that the properties of naturally or experimentally biased repertoires can be effectively harnessed for the generation of targeted human antibody libraries, substantially increasing the probability of isolating antibodies suitable for therapeutic and diagnostic applications.

## Introduction

A key characteristic of the adaptive immune system is its capacity to generate useful antibodies directed against invading pathogens. For this, the antibody repertoire expressed by B cells within an organism constantly evolves in response to infection. Phage, yeast, ribosome or bacterial display methodologies [1], [2] are employed extensively for the generation of antibodies to be used for research and therapeutic applications [3], [4]. This approach aims at recapitulating the process of appropriate antibody creation by the immune system, via *in vitro* selection of target-specific antibodies starting from a large repository of immunoglobulin genes [5].

Strategies for generating naive antibody repertoires – or libraries – capture different sources of diversity. In many cases, naturally rearranged antibody variable genes from animal or human donors are assembled to generate libraries based on natural diversity [6], [7], [8]. Alternatively, synthetic libraries are generated by introducing random diversity into the complementary determining regions (CDR) of specific antibody frameworks, the latter selected for stability and high frequency representation in human repertoires [9], [10], [11], [12]. A limitation of synthetic CDR diversification is that a significant proportion of randomized CDR sequences do not allow proper folding of the antibody variable region and, thus, reduce the number of functional members and overall performance of the library. This limitation can be partially mitigated by synthetic approaches where CDR sequences are designed to mimic natural diversity [11], [13], [14], [15]. One advantage of libraries based on naturally occurring sequences is that they include amino acid stretches in the CDR3 of the heavy chain (CDRH3) which are difficult to obtain with synthetic approaches. However, these libraries also contain variable domains that are less stable or under-represented in human repertoires. Both characteristics can increase the risk of being immunogenic and are, therefore, not desirable for the development of therapeutic antibodies. Another drawback of natural libraries that are based on variable genes, retrieved from circulating human B lymphocytes, is that these repertoires have been partially depleted for sequences reacting against self antigens and thus can be less effective for the isolation of antibodies targeting human proteins [16].

Regardless of the strategy that is used for construction, library size, diversity and functionality are important parameters that impact on the frequency and diversity of binders that can be obtained against an antigen of interest. Moreover, there is a clear correlation between library size and the affinity of the antibodies isolated [17]. Therefore, major efforts have been undertaken to generate very large naive libraries (i.e., in the range of 10^9^–10^11^ members) in order to identify antigen specific antibodies with affinities in the K_d_<10 nM range [7], [18], [19].

An alternative to large naive repertoires is the use of “immune” libraries based on biased antibody repertoires. These libraries incorporate rearranged variable regions from immunized animals [8], [20] or, in a limited number of cases, humans that suffer from cancer [21], have been exposed to pathogens [6], [22], [23] or show high antibody titers for a defined antigen [24]. High affinity and specific antibodies can be obtained from immune libraries as small as 10^5^ members [25], [26]. However, the need for immunization restricts the use of such libraries for therapeutic applications as animal derived antibodies trigger an immune response in patients, and naturally occurring immunization in humans is limited to very few targets.

The CDRH3 is the most diverse CDR in an antibody, both in length and amino acid sequence [27]. CDRH3 and CDRL3 (CDR3 of the light chain) form the center of the antigen combining site and analysis of antibody-antigen structures indicates that CDRH3 often significantly contributes to the interaction surface with the antigen [28], [29], [30]. Despite the CDRH3 high variability, trends in length and amino acid composition have been identified between species [31], [32]. In this study, the plasticity of antibody repertoires was exploited by capturing CDRH3 sequences from human and non-human sources and integrated into selected human frameworks. The result was the generation of human antibody libraries with characteristics of the sourced repertoires (i.e., length and amino acid composition profiles). Furthermore, using antigen biased repertoires, we could transfer their “immune” characteristics to human antibody libraries solely through the CDRH3. Our data validate an approach that enables efficient target specific library generation via exploiting the diversity of CDRH3 sequences found in different species under different conditions (naive versus immune) for a deeper and more complex starting point for generating therapeutic and diagnostic antibodies against challenging targets.

## Materials and Methods

### Ethics statement

This study was performed in accordance with the Swiss experimental animal regulations. The protocol was approved by the “Office Vétérinaire Cantonal de Genève” (Permit Number: 31.1.1015/3467/1). Every effort was made to minimize animals suffering.

### Reagents

All enzymes were provided by New England Biolabs and primers by Mycrosynth. PCR were performed with Go Taq from Promega and ligations with Rapid DNA ligation kit from Roche. M13KO7 helper phage, TG1 *E. coli* cells, pNDS vector and antigens (hIFNγ and hCCL5) were produced internally. TG1 cells were cultured in 2×TY media implemented with ampicillin 100 µg/ml (A - selection for pNDS vector), kanamycin 50 µg/ml (K - selection for helper phage vector) and glucose (G - repression of scFv production). For sequencing purposes, clones were cultured in LB supplemented with ampicillin and glucose (LB AG).

### Construction of the acceptor library

A library of pNDS phagemid vectors was generated including V genes coding for human heavy chain variable (VH) and light chain variable (VL) genes attached via a linker in a format of scFv. As described previously [33], 7 VH and 7 VL families were selected on the basis of their stability and frequency within the human antibody repertoire and extracted from genomes of Jurkat, Hela and HEK293 cells. At the location of the CDRH3, a non diversified out of frame “stuffer” sequence was inserted which included the recognition sites of BsmBI, a type IIS restriction enzyme. Synthetic diversity was incorporated at the location of the CDRL3 [33] while CDR1 and 2 of heavy and light chains remained germline. These scFv constructs were fused to a c-myc tag, a His tag and gIII for display on M13 bacteriophage. The expression and the plasmid replication were controlled by the lac operon and the pelB leader sequence. pNDS vector also possessed a gene of resistance to ampicillin allowing positive selection. This pNDS library was electroporated into TG1 *E. coli* cells generating a diversity of 2.2×10^9^ transformants. The library having the ability to accept CDRH3 sequences after BsmBI digestion, it was referred as the acceptor library.

### Immunization protocol

An immunization procedure was initiated for further exploration of naive and immune natural repertoires of CDRH3. Five BalbC mice (Janvier Laboratories) were kept naive while two groups of four BalbC mice were injected with hIFNγ (200 µg/mouse) or hCCL5 (400 µg/mouse) in RIBI adjuvant IP (intraperitoneal), IV (intravenous) and SC (subcutaneous) along three boosts and two hyperboosts (total of 55 days). Mice were sacrificed and their spleens recovered for RNA extraction. Serum antibodies titers were checked along the immunization procedure to ensure the development of a specific immune response against the antigen.

### Capture of murine CDRH3 encoding sequences

Spleens were treated on the day of sacrifice with ACK (NH4Cl 0.15 M, KHCO3 1 M, Na_2_EDTA 0.1 mM) in presence of DNAse (DN25 Sigma) and Collagenase (Type 4 from Invitrogen) and kept at −80°C in RNAlater (Ambion). mRNA was extracted from cells with RNAqueous kit (Ambion) and used as template for reverse transcription (RT) with Ready-to-Go You Prime beads (GE Healthcare). Murine CDRH3 genes were then amplified by nested PCR. Fifteen mouse VH families could be recovered along first amplification. The second was designed for the amplification of CDRH3 from the pool of VH and the incorporation of FokI recognition sites at both CDRH3 extremities. At the 3′ end, four specific primers were designed for J region sequences according to the IMGT database. At the 5′ end, a human primer was used in order to correct one base in murine sequences, which differs from human's, at the anticipated site of cleavage by FokI (Figure S1). The final DNA fragments obtained contained the natural mice CDRH3 corrected of one base at the 5′ end and surrounded by two FokI restriction sites. After each PCR, fragments were purified on E-Gel 2% (Invitrogen) and with the kit Wizard SV Clean-up System (Promega). Primers were also biotinylated for further purification.

### Capture of human CDRH3

Human CDRH3 from three donors were amplified by nested PCR from commercially available peripheral blood purified cells cDNA (AllCells and BioChain). The first PCR allowed the rescue of five main human VH families while a second PCR allowed for the amplification of only CDRH3 sequences. Similar to mouse diversity, primers incorporated a FokI recognition site and were biotinylated for purification purposes.

### Capture of in vitro biased CDRH3

Previously described AE1 library was used as a source of diversity [33], for clarity it will be referred as SnA. This library is based on the same design than the acceptor library except that synthetic diversity was introduced at the location of the CDRH3. After three rounds of phage display selection against hIFNγ, output was harvested and plasmid DNA extracted by Maxiprep (Promega). As described for murine diversity, synthetic CDRH3 were amplified by PCR with biotinylated primers which allowed the addition of FokI restriction sites at the extremities.

### Cloning of CDRH3 diversity into the acceptor library

The acceptor library and the CDRH3 inserts were respectively digested with BsmBI and FokI, two type IIS restriction enzymes generating four bases cohesive ends. The digested acceptor library DNA was purified on Chroma Spin T1000 columns (Clontech) for stuffer removal. CDRH3 were purified on Dynabeads (M280 Streptavidin from Invitrogen), for the removal of biotinylated primers and partially digested DNA fragments, followed by a phenol/chlorophorm purification step and a precipitation in ethanol. CDRH3 were then ligated into the acceptor library pool with Rapid DNA Ligation Kit (Roche) and electroporated into TG1 *E. coli* cells. Each library reached 10^7^ to 10^10^ transformants (Table 1).

**Table 1:** Summary of captured CDRH3 diversity and analysis of libraries by NGS.

CDRH3 Source	Library or Selection Round	Size	Sequencing		
			Total	Unique	%
Human	HnA	1.5×10^10^	7′878′344	2′448′285	31%
Murine	MnA	2.5×10^8^	2′539′098	604′929	24%
	MiB	7.3×10^7^	3′190′507	446′694	14%
	MiC	1.8×10^8^	2′951′698	575′107	19%
Synthetic	SnA	7.3×10^9^	5′078′705	5′007′022	99%
	SnA-R3	3.7×10^7^	1′247′375	67′936	5%
	SiB	1.1×10^9^	35′833′982	10′689′082	30%

HnA displays naive human CDRH3, MnA naive mice CDRH3, MiB CDRH3 from mice immunized with hIFNγ, MiC CDRH3 from mice immunized with hCCL5, SnA is the initial synthetic library, SnA-R3 is the selection round used as a source of diversity to generate SiB. The section “size” informs about the number of transformants after electroporation for the libraries and the output of selection for SnA-R3. Sequencing data show the total number of VH sequences analyzed by NGS and the number of unique VH they contained, also expressed as a percentage of the total sequences.

### Sequencing

Some clones were analyzed by classical Sanger sequencing. Individual clones were cultured in 2 mL of LB AG overnight and plasmid DNA was extracted by QIAprep Spin Miniprep Kit (Qiagen). DNA was then sequenced by Fasteris SA.

Libraries as well as rounds of selections were more thoroughly investigated by next generation sequencing (NGS) using the HiSeq Illumina platform provided by Fasteris SA (Switzerland). Primers initiating sequencing were designed in VH J region at the 3′ border of CDRH3, area common to all sequences. Procedure relative to the Illumina platform was described previously [33]. Sequencing, limited to 108 bp length, could cover CDRH3 and part of VH framework three. DNA signatures were used to identify framework families. Analysis of large data sets was performed with a software developed at NovImmune. Only the first 102 bp were analyzed to ensure minimum impact of sequencing errors which increase exponentially at the end of sequencing.

### Selection against hIFNγ

TG1 cells of each library were grown at 37°C and 240 rpm in 2×TY AG till OD 0.5. Libraries were then super-infected with M13K07 helper phage for 1 hr at 37°C (100 rpm) and medium replaced by 2×TY AK for the selection of bacteria having incorporated helper phage vector. Phage displaying scFv were expressed overnight at 30°C and 280 rpm and were then purified and concentrated by two precipitations in 1/3 v/v of 20% PEG-8000/2.5 M NaCl (Sigma), ultracentrifugation and dialysis in TE buffer. Phage preparations were titrated by infecting TG1 cells and reached around 10^13^ pfu/mL. A quantity of phage that covered diversity (10^10^ pfu for MnA, MiB and MiC; 10^12^ pfu for SnA and 4×10^11^ pfu for SiB) were blocked with 3% (w/v) skimmed milk in PBS. After two 1 h deselection steps on streptavidin coated magnetic beads (Invitrogen), phage were transferred to similar beads precoated with 100 nM biotinylated hIFNγ, for the actual selection to occur, during 2 h at room temperature. Non-specific phage were eliminated by five washes with PBS/0.1% Tween 20 and two washes with PBS. Remaining phage bound to beads were eluted with 10 mM triethylamine TEA (Sigma), neutralized with 1 M Tris-HCl pH7.4 (Sigma) and finally added to 10 mL of TG1 at OD 0.5 for 1 h infection at 37°C and 90 rpm. Resulting cells were titrated, spread on 2×TY AG agar bioassay plates and incubated overnight at 30°C. Colonies were then scraped off in 2×TY and stored in 17% glycerol at –80°C. For subsequent rounds of selection, 20 µL of cells from the preceding round were grown at 37°C and 240 rpm in 20 mL 2×TY AG 4% till OD 0.5, rescued with helper phage for 1 h at 37°C and 90 rpm, and medium was changed to 2×TY AK. After expression of phage overnight at 30°C and 280 rpm, 10 µL of supernatant was used as novel input. To define the amount of phage it represented, novel inputs were also titrated in TG1 cells. For each library three rounds of selection were performed against hIFNγ.

### ELISA Screening

TG1 colonies from each round of selection were individually picked and cultured in 96 wells plates for phage or scFv production. The binding properties of the scFv expressed alone or displayed on phage were then assessed by ELISA.

#### ScFv ELISA

Clones were cultured in 2×TY AG 2% for 6 hrs at 37°C and 130 rpm and IPTG (1 mM) was added for induction of scFv expression overnight at 30°C and 150 rpm. A positive control clone whose binding activity had been previously characterized was included on each plate. Maxisorb 96 well plates (Nunc) were coated with 1 µg/mL streptavidin (Roche) overnight at 4°C. The following day, scFv were blocked in 3% milk and streptavidin plates in 3% BSA. Biotinylated hIFNγ (1 µg/mL) was coated on half of Maxisorb plates. Plates coated only with streptavidin allowed to detect false positive. 50 µL of supernatants from each scFv plate were then transferred to a Maxisorb plates and incubated 2 h at room temperature. Bound ScFv were revealed with a mouse anti-c-myc antibody (produced internally) followed by a goat anti-mouse Fcγ HRP antibody (Jackson). After the addition of TMB substrate (Sigma) and blocking with H_2_SO_4_ (2N), absorbance at 450 nm was detected (Synergy HT from Bio TeK).

### Rescue of clones identified by NGS

Clones found to be strongly amplified by NGS and missed by screening were recovered by PCR. For each clone, overlapping primers were designed within the CDRH3 sequence. Two PCR were performed independently on selection round 3 to amplify, on one hand the beginning of the scFv till the CDRH3, and on the other hand the CDRH3 till the end of the scFv. Both fragments were then assembled by PCR, digested with NcoI and NotI, and ligated back into pNDS vector. Resulting clones were electroporated into TG1 and their sequence checked by Sanger sequencing.

### Dose response ELISA using purified scFv

Clones were cultured for periplasmic scFv production and were purified by Ni-NTA agarose chromatography (QIAgen) through their C-terminal His tag. Their concentration was determined by absorbance at 280 nm.

Maxisorb 96 well plates (Nunc) were coated overnight with streptavidin (Roche) and then blocked with 3% BSA. hIFNγ was coated at 1 µg/mL on one plate while a second plate was coated with only streptavidin and served as a negative control. Serial dilutions of scFv were then applied to both plates. After 2 h of binding at room temperature, previously described mouse anti c-myc and then goat anti mouse Fcγ HRP were applied followed by TMB for detection of scFv and H_2_SO_4_ for neutralization. Absorbance was read at 450 nm (Synergy HT from Bio TeK).

## Results

### Capture of natural CDRH3 diversity into human scFv libraries

**Figure 1:**
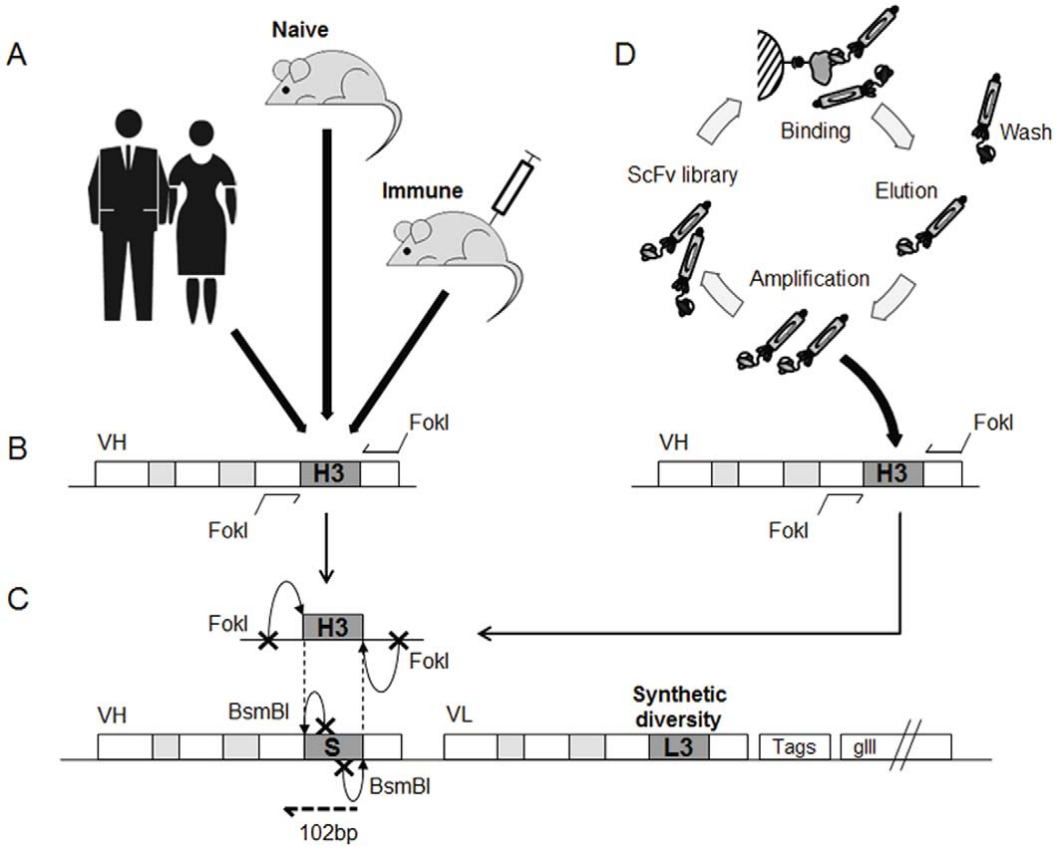
Strategy for capturing CDRH3 diversity into a human antibody library. (A) Natural diversity was incorporated from naive human donors and BalbC mice either kept naive or immunized with hIFNγ or hCCL5. (B) Their CDRH3 repertoires were extracted and amplified by PCR. During this step, recognition sites for the type IIS enzyme FokI were added. CDR3 are represented in dark grey, CDR1 and 2 in light grey. (C) Following digestion by FokI, repertoires of CDRH3 were cloned into an acceptor library itself digested with BsmBI. This second type IIS enzyme allowed the removal of a non diversified stuffer sequence (“S”) at the location of CDRH3 and the generation of compatible cohesive ends for the incorporation of CDRH3. The acceptor library combines 7 human VH and 7 human VL in a format of scFv as well as additional synthetic diversity in the CDRL3 (“L3” in the picture). Tags allow for purification while gIII allows for display on phage. After cloning of CDRH3, resulting libraries were analyzed by NGS covering the location of the CDRH3 and part of VH at the 5′ border (102 bp covered). (D) Similarly, sequences from a library of synthetic CDRH3, SnA, were explored in the context of the human acceptor library. Prior to the amplification, the repertoire of SnA was biased in vitro towards hIFNγ by performing three rounds of selection by phage display.

**Figure 2:**
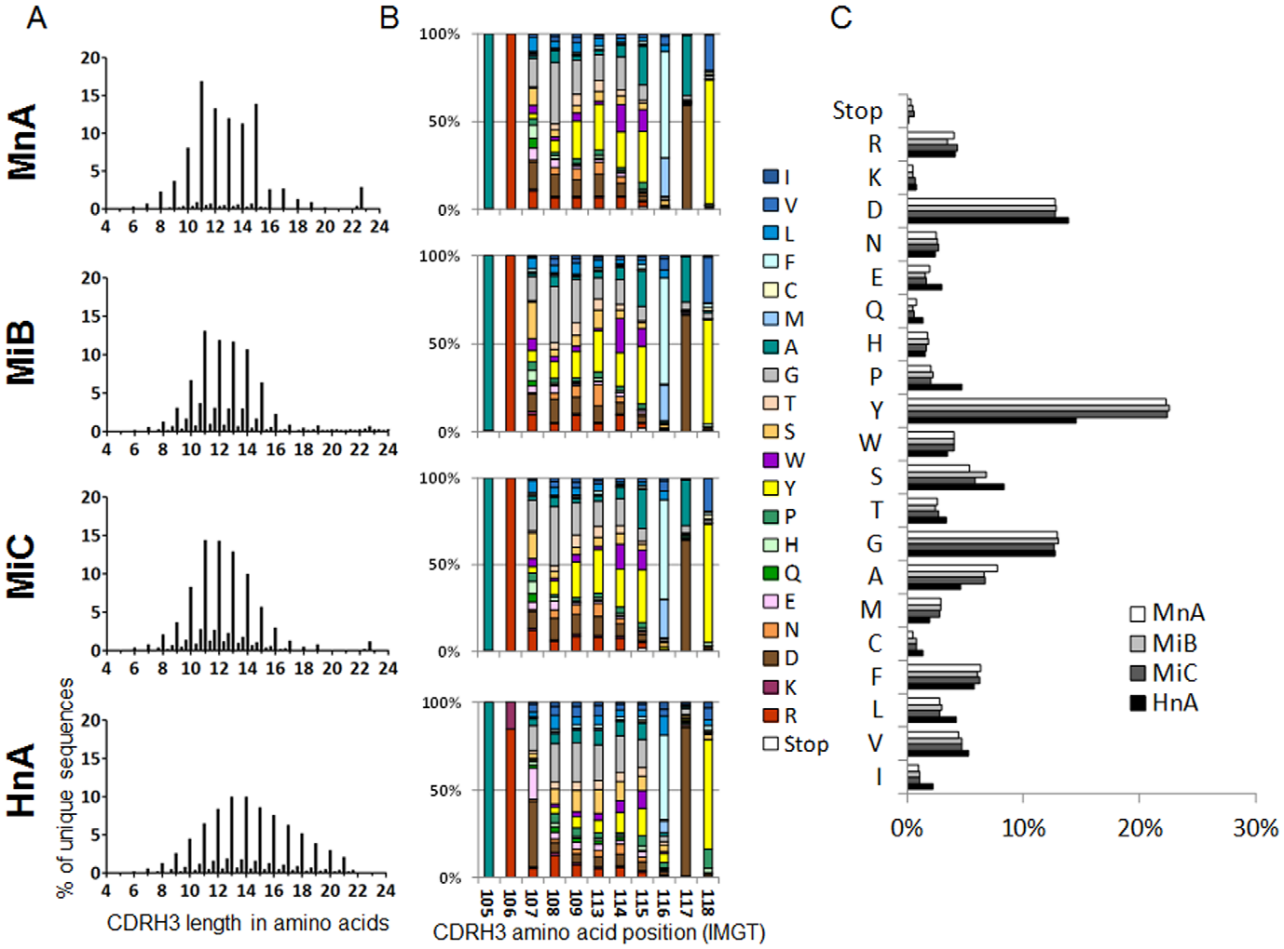
Characterization by NGS of captured murine and human CDRH3 repertoires. (A) Length profile of CDRH3 represented as the percentage of unique CDRH3 in function of their length in amino acids (AA). The minor peaks in between integers correspond to sequences where a frame shift occurred. (B) AA composition profile of unique CDRH3 of 11 AA, the most common length in murine diversity according to (A). For each position in the CDRH3, the percentage of AA was defined and illustrated according to a color code. IMGT numbering was followed to refer to AA positions. (C) AA composition profile of unique CDRH3 all lengths considered. The few stop codons found are likely due to cloning or NGS errors.

A method was established to retrieve and insert immunoglobulin CDRH3 sequences from different sources into the human heavy chain variable (VH) region, in order to exploit CDRH3 sequences derived from different natural immunoglobulin repertoires and in vitro antibody selection. Recently we described a cloning strategy using type IIS restriction enzymes for the insertion of CDR sequences into human antibody genes [33]. This approach relies on heavy and light chain acceptor frameworks that contain a stuffer DNA sequence, instead of the CDR3. The stuffer is removed by type IIS restriction enzyme digestion and replaced by CDR3 sequences that were encoded by synthetic oligonucleotides. This approach was modified to selectively amplify and clone, in a directional manner, CDRH3 sequences amplified from natural immunoglobulin repertoires. After a first amplification of VH genes using primers covering a variety of VH germline genes, the CDRH3 sequences were further amplified with primers specific for framework regions 3 and 4 (at the 5′ and 3′ borders of CDRH3, respectively), each containing a FokI restriction site (Figure 1). As the enzyme cleaves DNA 13 base pairs away from its binding site, the annealing of the primers to the target sequence is not affected by the enzyme recognition sequence. In this way, cohesive ends that are compatible with those of the BsmBI digested acceptor frameworks can be generated without modifying the framework or CDR coding sequence (Figure 1 A–C). CDRH3 sequences of human and murine origins were successfully incorporated (Figure 2) into human VH genes and combined with a light chain variable (VL) gene repertoire in which the CDRH3 had been diversified using synthetic sequences (Figure 1C).

As summarized in Table 1, a set of libraries were constructed with sizes ranging from 7.3×10^7^ to 1.5×10^10^. In the library HnA, CDRH3 sequences derived from healthy human donors were incorporated, whereas the libraries MnA, MiB and MiC were generated by inserting CDRH3 sequences obtained from mice into human VH genes. While human CDRH3 sequences are fully compatible with human acceptor frameworks, a single nucleotide difference, between the human acceptor frameworks and the mouse VH sequences at the 3′ boundary of the CDRH3, required a correction during the amplification process in order to generate compatible cohesive ends (Figure S1). The amplification and capture of murine CDRH3 sequences was performed using cDNA derived from splenocytes of naive mice (library MnA) or mice immunized with the human cytokine interferon gamma (hIFNγ) (library MiB), or the human chemokine hCCL5 (library MiC). Each animal had a specific IgG titer against the respective antigen prior to isolation of splenocytes (data not shown).

### Characterization of captured CDRH3 repertoires

The CDRH3 sequences that had been captured in the different libraries were characterized by next generation sequencing (NGS). As the library members have a common sequence in framework 4 (FR4), which is located after the CDRH3, a common sequencing primer was used, avoiding biases introduced by differences of annealing efficiencies when using multiple primers. The ability to read a length of 102 base pairs (bp) (Figure 1C) offered by the sequencing platform (Illumina) was sufficient to cover CDRH3 sequences of up to 24 amino acids in length and, in most cases, delivered sufficient framework 3 (FR3) sequence information to determine the VH subfamily used. A total of 7.9×10^6^, 2.5×10^6^, 3.2×10^6^ and 3.0×10^6^ sequences were obtained for the libraries HnA, MnA, MiB and MiC, respectively (Table 1). The analysis of CDRH3 lengths revealed that the HnA library contained a wider distribution and longer CDRH3 compared to the MnA, MiB and MiC libraries (Figure 2A). The amino acid composition was similar between the libraries containing murine sequences (Figure 2B and C). Of particular significance was the higher tyrosine content found in the MnA, MiB and MiC libraries in comparison to the HnA library. Shorter CDRH3 lengths and higher tyrosine content are hallmarks of murine CDRH3 sequences [31]. Thus, the NGS analysis indicated that human and mouse CDRH3 were captured into human VH frameworks and that human antibody libraries with CDRH3 having characteristics of human or murine repertoires could be successfully generated by this approach.

Further analysis revealed that HnA contained 31% unique sequences (i.e., 2.4×10^6^ were different VH sequences representing 31% of the total sequenced – 7.9×10^6^) (Table 1). Similarly, the unique sequences for the MnA, MiB and MiC libraries were 24%, 14% and 19% respectively. These results revealed a degree of redundancy of CDRH3 sequences in the libraries potentially due to biases introduced during the amplification steps. However, as the CDRH3 sequences are cloned into VH acceptor frameworks that are combined with diversified VL genes, each library member is likely to be unique. This hypothesis was confirmed by non high throughput di-deoxynucleotide terminated sequencing (i.e., Sanger) that provided information for both VH and VL genes. A limited number (>50) of scFv in each library were sequenced with this method and were all shown to be unique (data not shown). Interestingly, analysis of the frequency of repeated CDRH3 sequences showed that sequences with a certain degree of redundancy (i.e., sequences present more than 10^3^ times) represented 62%, 78% and 70% for MnA, MiB and MiC, respectively. In contrast, the number of non redundant sequences (identified only once) was 6%, 2% and 3% for MnA, MiB and MiC, respectively. This result suggests a trend for increased redundancy in the MiB and MiC libraries that incorporated sequences derived from immunized animals as compared to the naive MnA library (Figure S2A). This is consistent with a bias of the antibody repertoire, and in particular of CDRH3 sequences, towards the immunogen. The homogenous distribution of unique CDRH3 sequences into the different VH families indicated that no bias for one framework occurred during the cloning step (Figure S2B).

### Phage display selection using human antibody libraries containing murine CDRH3 repertoires

**Figure 3:**
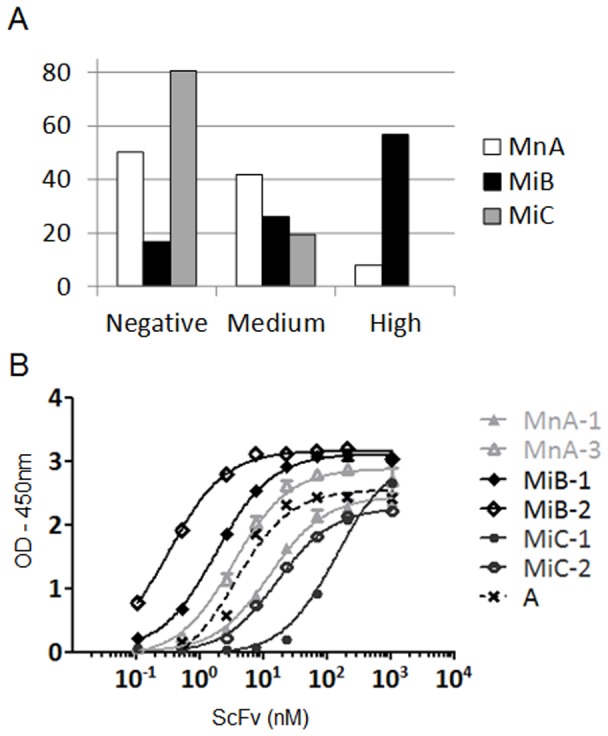
Screening of selections outputs from biased and naive murine libraries. (A) The supernatants of random clones from the selection round 3 against hIFNγ were tested independently in scFv ELISA against the same antigen (88 clones tested for each library). Clones were then ranked according to their level of absorbance at 450 nm. Were defined “high”, clones with absorbance values above 70% of the signal of a positive control scFv (absorbance ∼2.1), “medium”, clones between 10% and 70% (absorbance 0.3 and 2.1) and “negative”, clones below 10%. Histograms show the percentage of clones relative to their corresponding level of absorbance. (B) The two most frequent clones according to NGS were purified as scFv and tested in parallel in a dose response ELISA against hIFNγ (n = 2). Sequences from these clones are given in Table 2. The graph shows the level of absorbance at 450 nm in function of scFv concentration in nM. A is a positive control scFv.

In order to demonstrate that the CDRH3 repertoires that were isolated from mice and inserted into human VH frameworks could lead to the generation of functional antibodies, the MnA, MiB and MiC libraries were used in conjunction with phage display and selected against hIFNγ. After three rounds of selection, random clones were picked and tested as soluble scFv by ELISA for specific binding against the target (Figure 3A). The results showed a higher hit rate for MiB clones (83%) compared to the naive MnA library (50%) in turn higher compared to the irrelevant MiC library (19%). Further analysis revealed that amongst hits, the MiB library possessed an increased frequency of clones with high absorbance compared to MnA (clones with OD_450 nm_>2 represented 57% for MiB and 8% for MnA) for which the majority of clones displayed a medium level of absorbance (42% with 2>OD_450 nm_>0.3). The data indicate that the MiB library containing CDRH3 sequences isolated from mice that had been immunized with hIFNγ generated a significantly higher number of strong binders against hIFNγ as compared to the naive library, MnA. In contrast, the MiC library that contained CDRH3 sequences derived from mice immunized with hCCL5 performed even more poorly than MnA. To confirm these results, the ELISA was repeated using phage, which increased the overall signal due to avidity and signal amplification, demonstrating a similar relative performance of the three libraries (Figure S3 and Materials and Methods S1 A). We extended these findings by repeating the process using another target antigen. Mice were immunized with human IL6 receptor and biased libraries were generated and used in selections against hIL6 receptor as described above. The library generated with CDRH3 isolated from mice immunized with hIL6 receptor gave a higher hit rate by ELISA as compared to libraries generated using CDRH3 from naive mice or animals immunized with an irrelevant antigen (Figure S5 and Materials and Methods S1 B).

Sequence enrichment during the selection process was also followed by NGS. The most amplified sequences after the third round of selection (>0.3% of total sequences) for each library are shown in Table 2. The CDRH3 sequences of these enriched clones were used to design specific and complementary oligonucleotides to rescue complete scFv sequences by PCR [33]. The rescued scFv were expressed, purified and their EC_50_ for binding to hIFNγ determined by ELISA (Table 2). For those with frequencies above 1%, an EC_50_ in the range of 2.9 to 129.1 nM was measured. Non-binders were found at a frequency <1% in each library, and may have been enriched due to factors other than target binding during the selection process (e.g., such as favorable growth characteristics). The two most frequent clones (which represented the major output of each library, i.e., 90% of total sequences for MnA, 83% for MiB and 58% for MiC), were tested for binding to hIFNγ (Figure 3B). As the two most amplified clones from MnA (MnA-1 and MnA-2) encoded the same protein VH sequence, MnA-1 and 3 were tested. The most potent binders, MiB-1 and 2, originated from the library constructed with CDRH3 sequences isolated from mice immunized with the target used for the in vitro selection. Compared to MiB-1 and 2, MnA-1 and 3 were less potent, however, higher than that measured for MiC-1 and 2. As a result, the EC_50_ of the two most abundant sequences found in each library correlated with the performance of the three libraries.

**Table 2:** Frequency and potency of binders derived from murine diversity.

Clone ID	CDRH3	VH subfamily	VH Frequency	CDRL3	VL subfamily	Found in Screening	ScFv EC50 (nM)
MnA-1	ARGDAMDY	IGHV1-69	61.3%	GTWDDEPQNVV	IGLV1-51	Yes	15.9
MnA-2	ARGDAMDY	IGHV1-69	22.6%	GTWDGRGRLAV	IGLV1-51	Yes	18.0
MnA-3	ARDGYDWYFDV	IGHV3-30-3	5.7%	QQGWDGPPT	IGKV1-33	Yes	6.7
MnA-4	ARDAYDWYFDV	IGHV3-30-3	1.8%	QQGFDGPPT	IGKV1-33	Yes	14.7
MnA-5	ARDAWDWYFDV	IGHV3-30	0.8%	GTYDSGKLRV	IGLV1-51	No	No binding
MnA-6	ARRSGPYGAMDY	IGHV1-69	0.8%	QQQLGYRPPT	IGKV3-20	Yes	1.8
MnA-7	ARGDAIDY	IGHV1-69	0.3%	GTWDIGASYAV	IGLV1-51	No	349.4
MiB-1	ARSPLYWFFDV	IGHV3-30-3	58.8%	QQGTRRPTT	IGKV3-20	Yes	2.9
MiB-2	ARGYYGNPYYYAMDY	IGHV5-51	24.1%	QQGLYGPET	IGKV1-39	Yes	3.6
MiB-3	ARGDYGSRFAY	IGHV1-18	0.6%	AAYDGRLASV	IGLV1-44	No	71.0
MiB-4	ARSPLYWLFDV	IGHV3-30-3	0.6%	QQFGGRPTT	IGKV3-20	Yes	21.4
MiB-5	ARGTTVGGDYYPMDH	IGHV3-23	0.6%	QQRGAASPRT	IGKV1-33	No	1806.3
MiC-1	ARPDSLLYYWYFDV	IGHV3-23	47.1%	QQVAREPT	IGKV1-33	Yes	114.2
MiC-2	ARGHYGSSYYWYFDV	IGHV5-51	11.0%	AAWDENRPPV	IGLV1-44	Yes	22.2
MiC-3	ARHLYRAYAMDY	IGHV3-30	4.7%	QQELLTQPST	IGKV1-39	No	24.5
MiC-4	ARWGNYYRYDEAGKDAMDY	IGHV1-18	4.2%	QQRYPNPPWT	IGKV1-33	No	129.1
MiC-5	ARGDYDYAMDY	IGHV3-30-3	2.1%	QQLPVFPVT	IGKV1-39	Yes	4.5
MiC-6	ARSEYGAWFAY	IGHV1-2	0.9%	QQTATATPLT	IGKV3-11	No	No binding
MiC-7	ARHGYYAMDY	IGHV3-23	0.3%	QQQGHPAPKT	IGKV1-33	No	No binding

Information is provided on the MnA, MiB and MiC sequences of the most amplified VH after three rounds of selection against hIFNγ according to NGS with the corresponding percentages (from 0.3%). Most of clones with identical VH have been found by sequencing random clones with the Sanger method (referred as “found in screening”). Some others which had not been found were rescued by PCR from the output of selection. Information on VL sequences is then also described. The clones were purified as scFv and tested in dose response ELISA against hIFNγ, the last column shows the EC50 values observed in nM (n≥2).

In parallel to the NGS approach, the hits identified by ELISA screening were picked and their entire scFv sequence determined by the Sanger method (80 clones for MnA, 69 for MiB and 10 for MiC). All CDRH3 sequences had been found by NGS analysis. Conversely, some sequences identified by NGS with a frequency of up to 4.7% were not identified by the random screening ELISA approach. These sequences (MnA-5, MnA-7, MiB-3, MiB-5, MiC-3, MiC-4, MiC-6 and MiC-7) were rescued, expressed as scFv and evaluated in the ELISA (Table 2). Five of eight bound specifically to hIFNγ, further illustrating that NGS is a powerful and complementary approach for antibody discovery [33], [34], [35]. It is also interesting to note that the diversity is higher in the MiB library where all of the selected CDRH3 sequences are different (Table 2), in contrast to the MnA library in which the enriched sequences can be clustered into three groups of identical or very similar sequences (one amino acid change). A wider diversity of VH subfamilies was also isolated from the MiB library (Table 2).

### Capture of synthetic CDRH3 repertoires enriched in vitro

**Figure 4:**
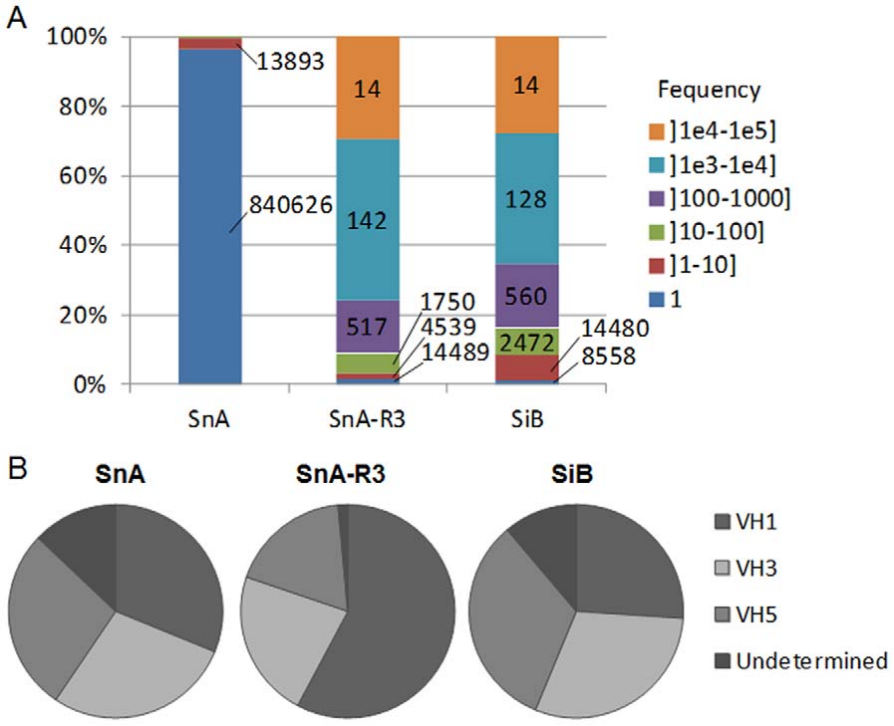
Analysis of synthetic CDRH3 diversity in SnA, SnA-R3 and SiB. Evaluation by NGS of the diversity of synthetic CDRH3 from SnA, SnA-R3 and SiB (one million sequences were analyzed for each) and their repartition into human frameworks. (A) The percentage of CDRH3 in function of their frequency is represented by a color code. The values on the histograms are the number of unique CDRH3 corresponding to each section. (B) Frameworks repartition by family VH1, 3 or 5 in each library and in the selection round SnA-R3. Undetermined are sequences for which no framework could be attributed due to frame shifts.

As the MiB library led to increased hit rates and clones with higher diversity and apparent affinity compared to a naive library or a library biased against an irrelevant target, we then reasoned that capturing CDRH3 after in vitro selection against a given target would also provide a source of diversity to generate biased libraries. To test our hypothesis, a previously described [33] naive synthetic scFv library of 7.3×10^9^ transformants (SnA) was used in selection against hIFNγ. Phagemid DNA was extracted from bacteria infected with phage obtained after a third round of selection (called SnA-R3) and used for CDRH3 amplification by a two step PCR approach (Figure 1D). The amplified CDRH3 sequences were digested with FokI and cloned into acceptor VH frameworks to generate a new library of 1.1×10^9^ transformants, referred to as SiB (Table 1). The SnA library, the output of selection round SnA-R3 and the newly generated SiB library were analyzed by NGS. A total of 5.0×10^6^, 1.2×10^6^ and 3.6×10^7^ sequences covering the CDRH3 were analyzed for SnA, SnA-R3 and SiB, respectively. As expected for a library constructed using synthetic oligonuclotides, SnA featured a high level of diversity as 99% of its CDRH3 sequences were unique. The selection process against the target led to an enrichment of sequences (5% VH diversity in SnA-R3) especially significant for certain sequences as 14 CDRH3 represented 29.5% of the total (Figure 4A). This biased CDRH3 repertoire was efficiently transferred into the SiB library as the frequency of CDRH3 sequences was similar between the two sets (Figure 4A). The proportion of unique VH sequences in SiB rose to 30% as CDRH3 sequences were cloned into the context of different VH germlines (Table 1). These results were confirmed by the analysis of the distribution of VH families (Figure 4B). The distribution was relatively equivalent between VH1, VH2 and VH5 families in the SnA library. After selection, a bias for VH1 was observed in SnA-R3, whereas the distribution after recloning into the SiB library was, as expected, again relatively even (Figure 4B). The CDRH3 sequences were indeed redistributed after cloning in different VH germlines. CDRH3 could also be associated to a variety of VL, however, this diversity could not be evaluated by NGS. Sequences for which the VH subfamily could not be determined were classified as undetermined (Figure 4B). These sequences reached 13% for SnA and 11% for SiB, whereas only 2% of undetermined frameworks where found in SnA-R3. The higher frequencies observed in the libraries are likely due to cloning problems leading to frame shifts as potential sequencing errors should be equivalent in all samples. In addition, a lower frequency of undetermined frameworks in SnA-R3 is in line with this observation as non functional sequences are lost during selection rounds.

### Performance of libraries biased via in vitro methods of selection

**Figure 5:**
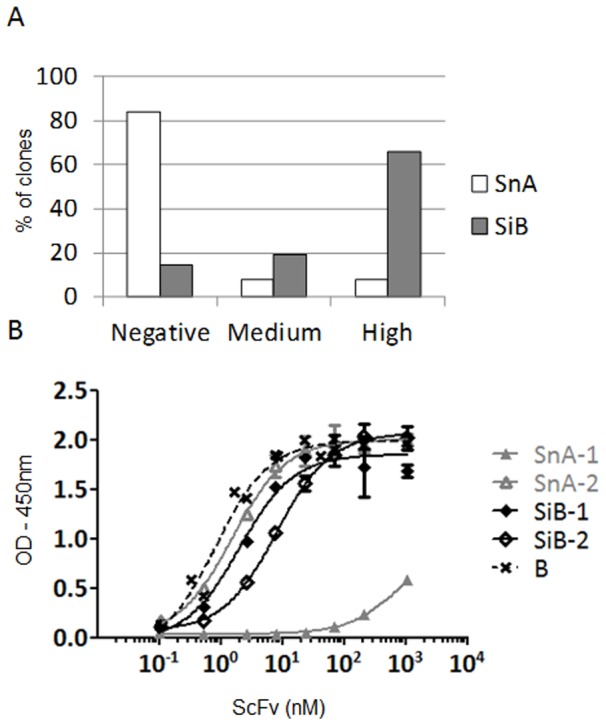
Screening of selections outputs from biased and naive synthetic libraries. (A) The supernatants of random clones from the selection round 3 against hIFNγ were tested independently in scFv ELISA against the same target (88 clones tested for each library). Clones were then ranked according to their level of absorbance at 450 nm. Were defined “high”, clones with absorbance values above 70% of the signal of a positive control scFv (absorbance ∼1.8), “medium”, clones between 10% and 70% (absorbance between 0.3 and 1.8) and “negative”, clones below 10%. Histograms show the percentage of clones relative to their corresponding level of absorbance. (B) The two most frequent binders according to NGS were purified as scFv and tested in parallel in a dose response ELISA against hIFNγ (n = 2). Sequences from these clones are given in Table 3. The graph shows the level of absorbance at 450 nm in function of scFv concentration in nM. B is a positive control.

Both SnA and SiB libraries were used in parallel in phage display selections against hIFNγ. After three rounds of selection, the output was evaluated with scFv by ELISA on randomly selected clones. Similarly to the MiB library, the frequency of positive clones and, in particular, of clones giving rise to a strong binding was much higher in the output of the SiB library as compared to the SnA library. The majority (66%) of clones derived from the SiB library displayed strong binding, while the majority (84%) of clones derived from the SnA library were found to be non binders (Figure 5A). ELISA on the same clones was also performed using phage and confirmed the scFv ELISA results, as 100% of clones derived from the SiB displayed a high absorbance while 82% of SnA derived clones remained negative (Figure S4 and Materials and Methods S1 A). Clones identified in both libraries were sequenced to determine their diversity. Ten and eleven unique scFv were identified from the SnA and SiB libraries, respectively (Table 3). Identical CDRH3 sequences were found in the scFv SnA-3 and SiB-7 and in the scFv SnA-7 and SiB-5. In both cases, these CDRH3 were found in the same VH germline context but were combined with VL sequences having different CDRL3 sequences. The other CDRH3 sequences were different, varying in length and sequence. As expected, all CDRH3 of the SiB clones defined in Table 3 were found by NGS in SnA-R3 where their frequencies reached 0.03 to 1.36%. Each unique scFv was expressed and tested in a dose-response ELISA against hIFNγ in order to determine an EC_50_ (Table 3, Figure 5B). The EC_50_ value ranged from 1.1 to 894.9 nM and 0.8 to 31.2 nM for SnA and SiB, respectively. The CDRH3 frequency of third round of selection outputs were also analyzed by NGS (Table 3). The most frequent CDRH3 sequence (3.7%) derived from the SnA library corresponded to the SnA-1 scFv with an EC_50_ of 895 nM. In contrast, the most frequent CDRH3 form of the SiB output (42.5%) corresponded to the scFv SiB-1 having an EC_50_ of 2 nM. These results indicate that the retrieval of high affinity clones is more frequent when a scFv library incorporating a CDRH3 repertoire previously biased in vitro against hIFNγ is generated and used. Moreover, novel candidates, not found in the non biased synthetic library, were identified. Using this approach thus allowed for a more extensive sampling of diversity.

**Table 3:** Frequency and potency of binders derived from synthetic diversity.

Clone ID	CDRH3	VH subfamily	VH Frequency	CDRL3	VL subfamily	ScFv EC50 (nM)
SnA-1	ARETDSWDTFDY	IGHV1-69	3.74%	AAWDGASRRVV	IGLV1-44	894.9
SnA-2	ARYSNHPNASYMDY	IGHV5-51	0.66%	GTYDDMLQSFV	IGLV1-51	1.5
SnA-3	ARYNSHPHSPYMDY •	IGHV5-51	0.27%	GTWDMQQGAMV	IGLV1-51	6.8
SnA-4	ARGYWSASFDY	IGHV1-69	0.23%	AAYDGLGHVV	IGLV1-44	39.3
SnA-5	ARGSGYYTAGSFDY	IGHV5-51	0.21%	AAYDDATQAPV	IGLV1-44	25.8
SnA-6	ARGGWGASFDY	IGHV1-69	0.19%	AAYDGAGGVV	IGLV1-44	1.1
SnA-7	ARYTYHPDGGDMDY Δ	IGHV5-51	0.18%	GTWDESRSWV	IGLV1-51	4.1
SnA-8	ARGAGMDY	IGHV1-18	0.11%	AAWDDAQTSPV	IGLV1-44	109.3
SnA-9	ARSWWEDGSFDY	IGHV1-69	0.10%	GTYDKSNRGVV	IGLV1-51	17.2
SnA-10	ARESYAGKMDY	IGHV3-23	0.04%	QQSGLDPWT	IGKV1-39	1.9
SiB-1	ARGHDRRSGDFDY	IGHV5-51	42.52%	GTYDMGVSGTV	IGLV1-51	2.0
SiB-2	ARYTYHPTAPNFDY	IGHV5-51	6.16%	GTYDRLPAFLV	IGLV1-51	8.1
SiB-3	ARGYGHYHAGAFDY	IGHV5-51	2.80%	AAYDGAWGNAV	IGLV1-44	6.0
SiB-4	ARGDAMDY	IGHV1-69	1.50%	GTWDIGYSSVV	IGLV1-51	31.2
SiB-5	ARYTYHPDGGDMDY Δ	IGHV5-51	1.35%	GTYDAPDAYV	IGLV1-51	13.8
SiB-6	ARWNYWSGDMDY	IGHV5-51	1.03%	GTWDDSEAEV	IGLV1-51	9.7
SiB-7	ARYNSHPHSPYMDY •	IGHV5-51	0.68%	GTWDGRGTVFV	IGLV1-51	10.1
SiB-8	ARGWWDARFDY	IGHV1-69	0.43%	AAYDGWGTPV	IGLV1-44	2.2
SiB-9	ARYAAWWEGMDY	IGHV5-51	0.29%	GTYDKKPSVLV	IGLV1-51	0.8
SiB-10	ARYSYHPSAGSFDY	IGHV5-51	0.15%	GTYDTGPERPV	IGLV1-51	6.4
SiB-11	ARNKWASWSMDY	IGHV1-69	0.11%	GTYDVRQRNMV	IGLV1-51	27.3

Information is provided on the SnA and SiB sequences found by screening positive clones in scFv ELISA after three rounds of selection against hIFNγ. Corresponding VH frequencies by NGS are described. Full circles and white triangles highlight identical CDRH3 in both SnA and SiB groups. Clones were purified as scFv and tested in dose response ELISA against hIFNγ, the last column shows the EC50 values observed in nM (n = 2).

To ensure specificity of the scFv sequences, three of the most amplified clones from each library (naive, biased in vivo and in vitro) were tested at different concentrations for binding to hIFNγ and a panel of 8 irrelevant targets. All the scFvs were specific for hIFNγ and did not show any significant unspecific binding (Figure S6 and Materials and Methods S1 C).

## Discussion

Immunoglobulin repertoires are very plastic and B cells populations constantly evolve in response to different pathogens and other stimuli. In addition, the sequences encoding immunoglobulins that recombine during B cell development to generate functional antibodies differ between species. Several studies have shown that CDR sequences of non-human origin can be used to generate humanized antibodies [36], [37], [38]. The aim of our study was to isolate and capture the features of immunoglobulin repertoires from different species or from B cell populations that have been biased in response to antigens and incorporate these features into human antibody libraries. We developed a generic approach to retrieve CDRH3 sequences from different sources and clone them into selected human antibody frameworks using type IIS restriction cloning that avoids modification of the antibody coding sequence either in the CDRs or in the antibody framework regions. Although applicable to any CDR, we focused on the CDR3 of the heavy chain as it is the most diversified region of an antibody and in many cases contributes to most of the antigen binding energy [28], [29].

Five phage antibody libraries were constructed by capturing CDRH3 sequences from human healthy donors or mice that were either naive or immunized with different antigens. We then analyzed these libraries by NGS which is a powerful method to extensively characterize such large repertoires [33], [35]. The analysis showed that the new libraries contained CDRH3 having length and amino acid compositions typical of murine or human CDRH3 sequences, demonstrating that CDR capture enables the generation of human antibody libraries that probe three dimensional space differently. This might allow taking advantage of long CDR loops found in camelid VHH or shark V-NAR domains that are much better suited to target cavities such as enzyme active sites or difficult to access epitopes in canyons of viruses [39], [40]. Furthermore, by cloning CDR sequences into selected frameworks, the probability of generating a functional antibody in the new context is higher compared to the use of synthetically diversified CDRs that frequently lead to non-functional antibodies that cannot fold properly. In this way, the benefit of using stable frameworks and CDR sequences that have been proofread in another repertoire can be combined for improved library functionality.

It has been demonstrated that even small antibody libraries constructed by combinatorial assembly of heavy and light chain variable regions derived from immunized animals facilitate the isolation of specific antibodies against the antigen used as immunogen [8], [20]. This is explained by the fact that recombining a VH and VL repertoire enriched for antigen specific sequences leads to more frequent productive combinations in the library and, thus, increased hit rates and binding affinities. In contrast, naive repertoires based on assembly of variable genes from naive sources, need to be of a much larger size (>10^9^) in order to have reasonable chances of success [41].

We reasoned that by capturing the central element of diversity of a biased antibody repertoire and grafting it into a novel antibody library, we could transfer its target specificity. This hypothesis was demonstrated using two independent sources of biased CDRH3 sequences. First, three libraries incorporating CDRH3 isolated from naive or immunized mice were used for phage display selections. These libraries had identical characteristics in terms of variable gene family content but led to significantly different performances both in term of hit rate and target binding capacity of the purified antibody fragments. This first approach demonstrated that for two model antigens, hIFNγ and hIL6 receptor, the target specificity of an in vivo biased repertoire could be transferred via its CDRH3 content.

We extended and confirmed these finding by using as a source of CDRH3 sequences, the output of a third round of phage selection against hIFNγ. Despite its smaller size, the biased library more frequently generated clones having higher binding capacity against hIFNγ compared to a larger naive library. Libraries especially designed to address a certain class of antigen have been difficult to generate and only few examples have reported for instance against haptens [42].

In this study, we describe an efficient approach to generate antigen targeted human antibody libraries that do not require immunized human donors and can be useful to address difficult targets. In summary, capturing CDR sequences allows in a simple and efficient manner to harness evolving immunoglobulin repertoires to generate novel and tailor made antibody libraries for in vitro selection of human antibodies.

## Supporting Information

Figure S1 **Difference between mouse and human DNA sequences at the 5′ boundary of CDRH3.** DNA sequences from natural murine IgG (source IMGT) and from the human acceptor library at the border of CDRH3. The underlined bases correspond to the cohesive ends generated after digestion by respectively FokI for murine sequences and BsmBI for the human acceptor library. At the 5′ border, one base systematically differs between mouse and human preventing efficient cloning. This base was then corrected by PCR along amplification of BalbC mice CDRH3. FW stands for framework.
(TIF)

Figure S2 **Analysis of CDRH3 diversity in MnA, MiB and MiC.** Evaluation by NGS of the diversity of murine CDRH3 in the context of MnA, MiB and MiC and their repartition into human frameworks (2.5, 3.2 and 3.0 million sequences analyzed, respectively). (A) Percentage of CDRH3 in function of their frequency represented by a color code. The values on the histograms are the number of unique CDRH3 corresponding to each section. (B) Frameworks repartition by family VH1, 3 or 5 in each library. Undetermined are sequences for which no framework could be attributed due to frame shifts.
(TIF)

Figure S3 **Screening of selections outputs from biased and naive murine libraries in phage format.** The supernatants of random clones from the selection round 3 against hIFNγ were tested independently in phage ELISA against the same target (88 clones tested for each library). Clones were then ranked according to their level of absorbance at 450 nm. Were defined “high”, clones with absorbance values above 70% of the signal of a positive control scFv (absorbance ∼1.5), “medium”, clones between 10% and 70% (absorbance between 0.2 and 1.5) and “negative” clones below 10%. Histograms show the percentage of clones relative to their corresponding level of absorbance. See also Materials and Methods S1 A.
(TIF)

Figure S4 **Screening of selections outputs from biased and naive synthetic libraries in phage format.** The supernatants of random clones from the selection round 3 against hIFNγ were tested independently in phage ELISA against the same target (88 clones tested for each library). Clones were then ranked according to their level of absorbance at 450 nm. Were defined “high”, clones with absorbance values above 70% of the signal of a positive control (absorbance ∼1.1), “medium”, clones between 10% and 70% (absorbance between 0.2 and 1.1) and “negative”, clones below 10%. Histograms show the percentage of clones relative to their corresponding level of absorbance. See also Materials and Methods S1 A.
(TIF)

Figure S5 **Screening of selections outputs from biased and naive murine libraries in phage format – Second example.** The supernatants of random clones from the selection round 2 against hIL6 receptor were tested independently in phage ELISA against the same target (88 clones tested for each library). Clones were then ranked according to their level of absorbance at 450 nm. Were defined “high”, clones with absorbance values above 70% of the signal of a positive control scFv (absorbance ∼1.6), “medium”, clones between 10% and 70% (absorbance between 0.2 and 1.6) and “negative” clones below 10%. Histograms show the percentage of clones relative to their corresponding level of absorbance. See also Materials and Methods S1 B.
(TIF)

Figure S6 **Specificity ELISA.** (A) Fifteen scFv isolated from the different libraries were tested in ELISA at three different concentrations (1100, 110 and 11 nM, n = 2) against hIFNγ and a panel of irrelevant targets, i.e. streptavidin, lysozyme, three human IgGs (VH3/Vλ6, VH4/Vκ6, VH1/Vλ3), horseradish peroxidase (HRP), viral CC-chemokine inhibitor (vCCI) and mouse toll like receptor 4 (mTLR4). When HRP was used as a target, revelation was performed via alkaline phosphatase and absorbance was read at 405 nm, for all the other targets, revelation was performed via HRP and absorbance was read at 450 nm. (B) Proper coating of all proteins used as targets was confirmed with specific antibodies (n = 2). See also Materials and Methods S1 C.
(TIF)

Materials and Methods S1 **Materials and methods** **for supporting figures.** Materials and methods are described for (A) the screening phage ELISA, (B) the second example of CDRH3 biased in vivo and (C) the specificity ELISA.
(DOCX)
