# Supplementary figures and images for: Transferring the Characteristics of Naturally Occurring and Biased Antibody Repertoires to Human Antibody Libraries by Trapping CDRH3 Sequences
Source: PLoS One. 2012 Aug 24;7(8):e43471. doi: 10.1371/journal.pone.0043471 (PMC3427355; doi:10.1371/journal.pone.0043471)

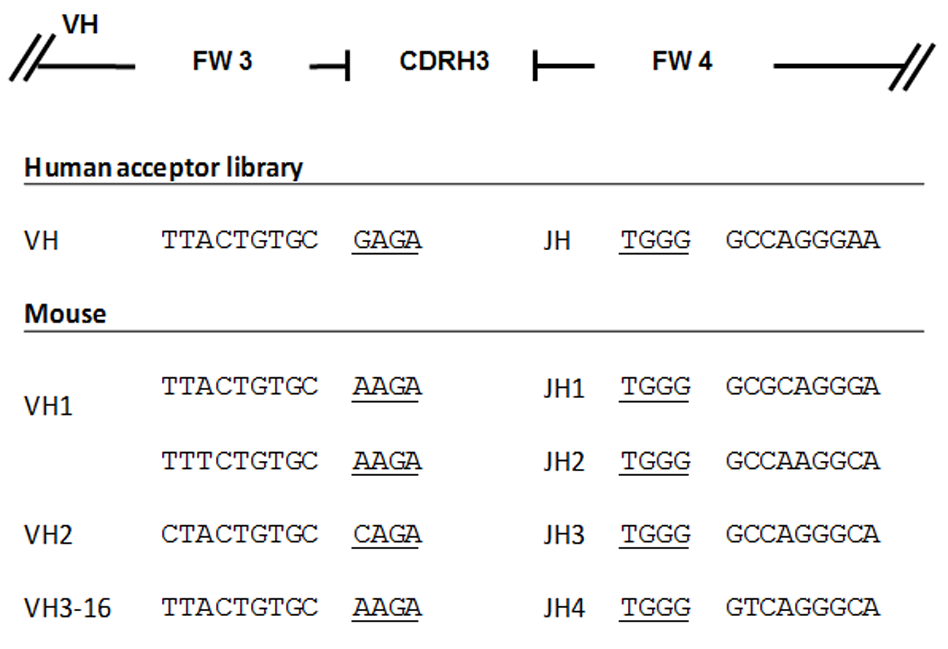

Supplement: Figure S1 — Difference between mouse and human DNA sequences at the 5′ boundary of CDRH3. DNA sequences from natural murine IgG (source IMGT) and from the human acceptor library at the border of CDRH3. The underlined bases correspond to the cohesive ends generated after digestion by respectively FokI for murine sequences and BsmBI for the human acceptor library. At the 5′ border, one base systematically differs between mouse and human preventing efficient cloning. This base was then corrected by PCR along amplification of BalbC mice CDRH3. FW stands for framework. (TIF) [file pone.0043471.s001.tif]

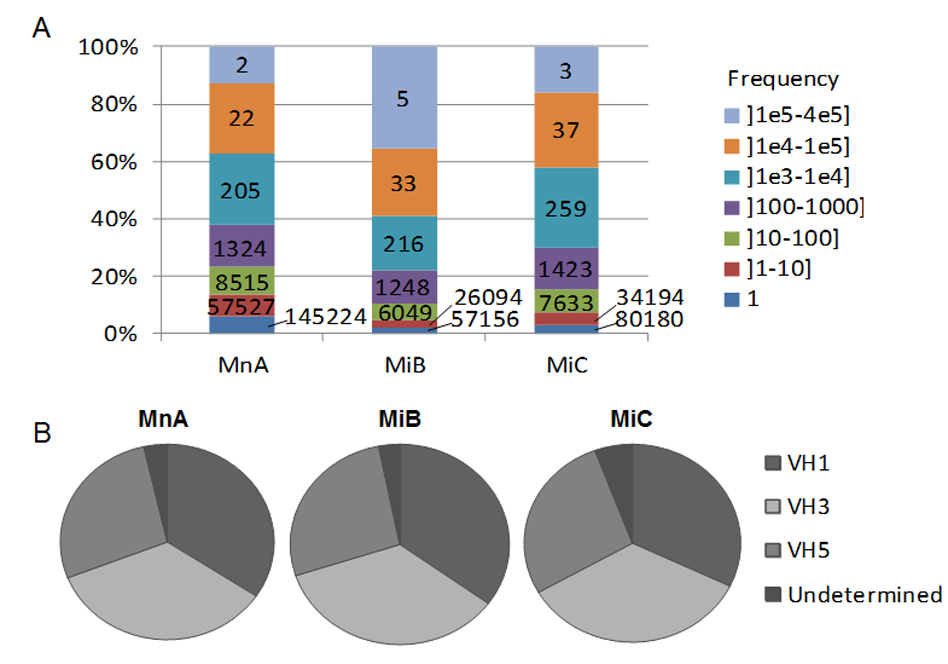

Supplement: Figure S2 — Analysis of CDRH3 diversity in MnA, MiB and MiC. Evaluation by NGS of the diversity of murine CDRH3 in the context of MnA, MiB and MiC and their repartition into human frameworks (2.5, 3.2 and 3.0 million sequences analyzed, respectively). (A) Percentage of CDRH3 in function of their frequency represented by a color code. The values on the histograms are the number of unique CDRH3 corresponding to each section. (B) Frameworks repartition by family VH1, 3 or 5 in each library. Undetermined are sequences for which no framework could be attributed due to frame shifts. (TIF) [file pone.0043471.s002.tif]

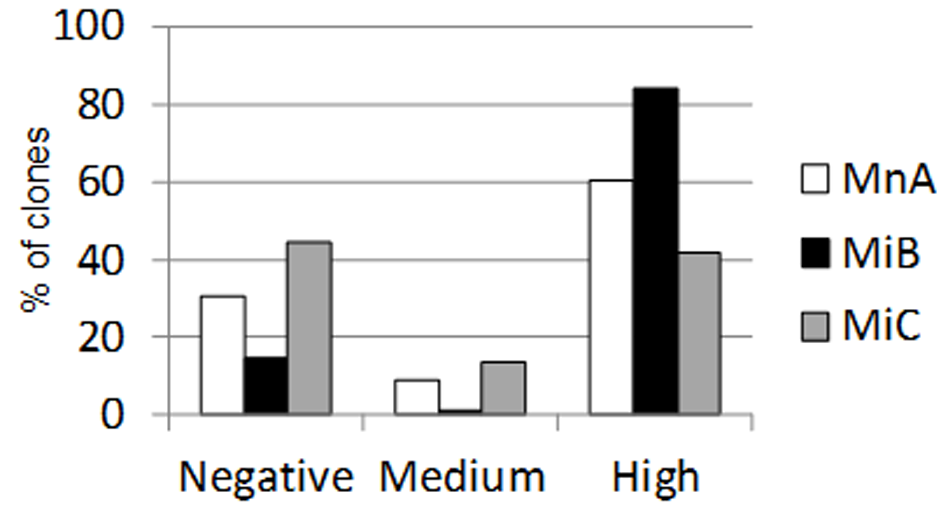

Supplement: Figure S3 — Screening of selections outputs from biased and naive murine libraries in phage format. The supernatants of random clones from the selection round 3 against hIFNγ were tested independently in phage ELISA against the same target (88 clones tested for each library). Clones were then ranked according to their level of absorbance at 450 nm. Were defined “high”, clones with absorbance values above 70% of the signal of a positive control scFv (absorbance ∼1.5), “medium”, clones between 10% and 70% (absorbance between 0.2 and 1.5) and “negative” clones below 10%. Histograms show the percentage of clones relative to their corresponding level of absorbance. See also Materials and Methods S1 A. (TIF) [file pone.0043471.s003.tif]

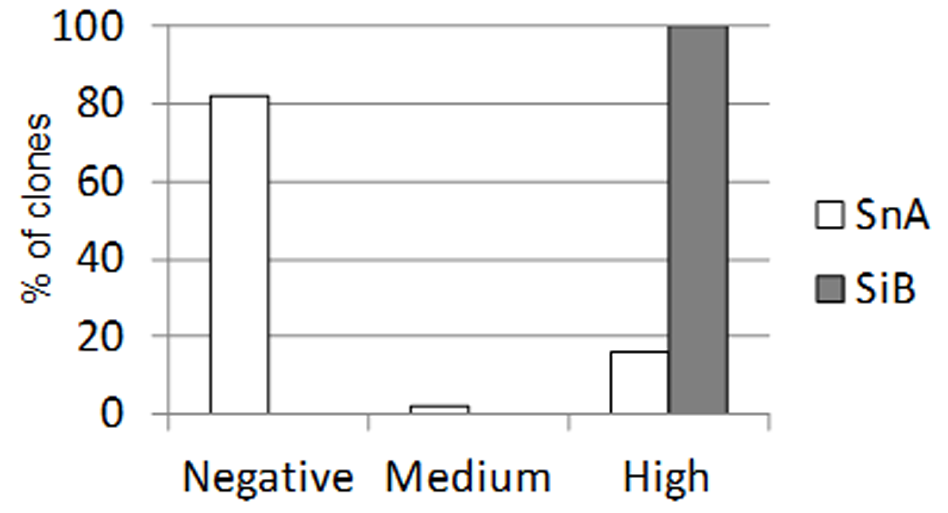

Supplement: Figure S4 — Screening of selections outputs from biased and naive synthetic libraries in phage format. The supernatants of random clones from the selection round 3 against hIFNγ were tested independently in phage ELISA against the same target (88 clones tested for each library). Clones were then ranked according to their level of absorbance at 450 nm. Were defined “high”, clones with absorbance values above 70% of the signal of a positive control (absorbance ∼1.1), “medium”, clones between 10% and 70% (absorbance between 0.2 and 1.1) and “negative”, clones below 10%. Histograms show the percentage of clones relative to their corresponding level of absorbance. See also Materials and Methods S1 A. (TIF) [file pone.0043471.s004.tif]

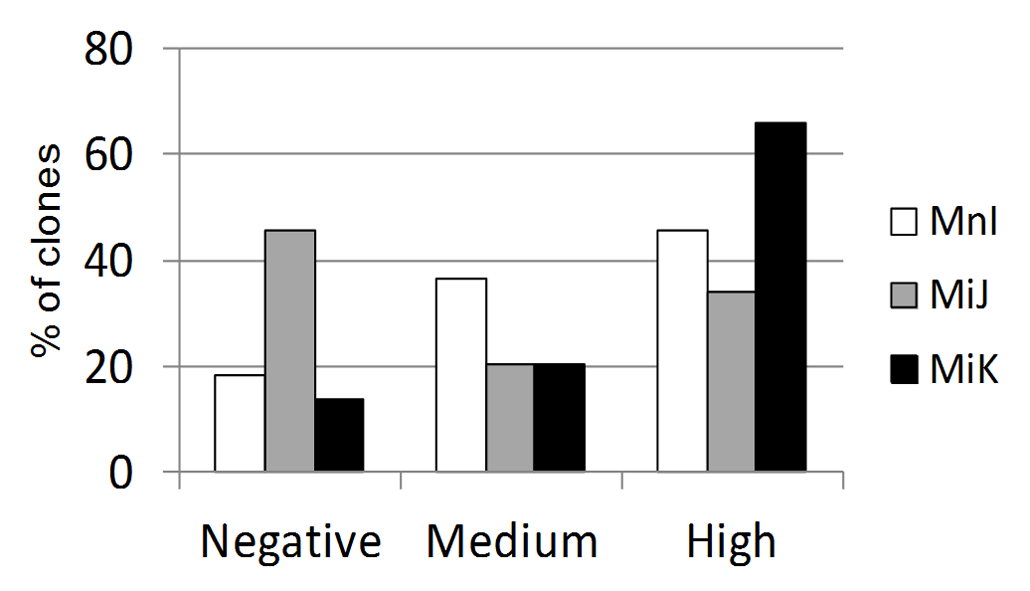

Supplement: Figure S5 — Screening of selections outputs from biased and naive murine libraries in phage format – Second example. The supernatants of random clones from the selection round 2 against hIL6 receptor were tested independently in phage ELISA against the same target (88 clones tested for each library). Clones were then ranked according to their level of absorbance at 450 nm. Were defined “high”, clones with absorbance values above 70% of the signal of a positive control scFv (absorbance ∼1.6), “medium”, clones between 10% and 70% (absorbance between 0.2 and 1.6) and “negative” clones below 10%. Histograms show the percentage of clones relative to their corresponding level of absorbance. See also Materials and Methods S1 B. (TIF) [file pone.0043471.s005.tif]

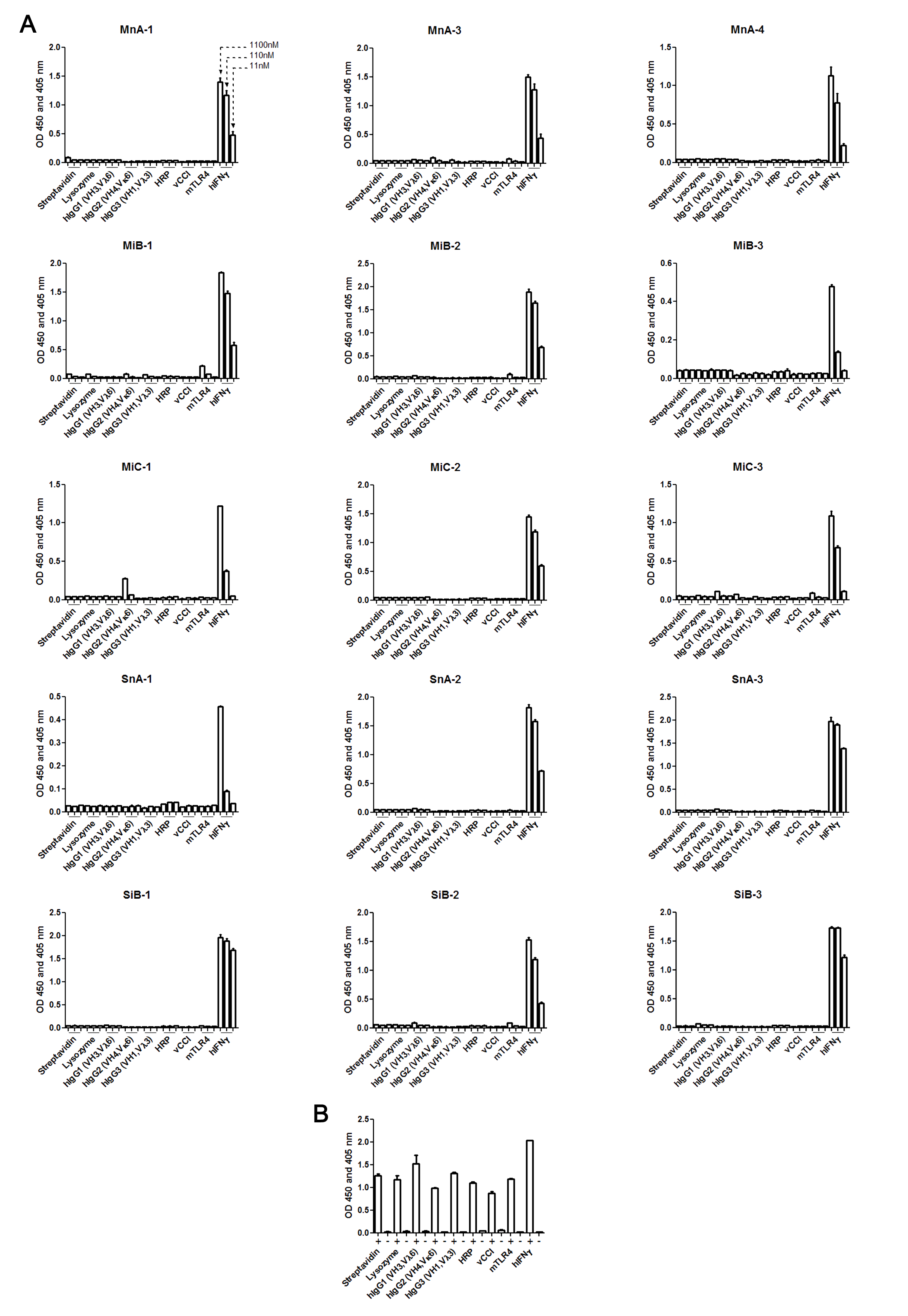

Supplement: Figure S6 — Specificity ELISA. (A) Fifteen scFv isolated from the different libraries were tested in ELISA at three different concentrations (1100, 110 and 11 nM, n = 2) against hIFNγ and a panel of irrelevant targets, i.e. streptavidin, lysozyme, three human IgGs (VH3/Vλ6, VH4/Vκ6, VH1/Vλ3), horseradish peroxidase (HRP), viral CC-chemokine inhibitor (vCCI) and mouse toll like receptor 4 (mTLR4). When HRP was used as a target, revelation was performed via alkaline phosphatase and absorbance was read at 405 nm, for all the other targets, revelation was performed via HRP and absorbance was read at 450 nm. (B) Proper coating of all proteins used as targets was confirmed with specific antibodies (n = 2). See also Materials and Methods S1 C. (TIF) [file pone.0043471.s006.tif]
