# Supplementary material for: Transferring the Characteristics of Naturally Occurring and Biased Antibody Repertoires to Human Antibody Libraries by Trapping CDRH3 Sequences
Source: PLoS One. 2012 Aug 24;7(8):e43471. doi: 10.1371/journal.pone.0043471 (PMC3427355; doi:10.1371/journal.pone.0043471)
Supplement: Materials and Methods S1 — Materials and methods for supporting figures. Materials and methods are described for (A) the screening phage ELISA, (B) the second example of CDRH3 biased in vivo and (C) the specificity ELISA. (DOCX) [file pone.0043471.s007.docx]

**MATERIALS AND METHODS S1**

**(A) Screening phage ELISA.**

Clones were cultured in 2xTY AG 4% for 6 h at 37°C and 130rpm and then infected with helper phage for 1h at the same temperature. Medium was then changed to 2xTY AK for overnight expression of phage at 30°C and 150rpm. A procedure similar to scFv ELISA was then performed except that a mouse anti M13-HRP (Amersham) antibody was used for the detection.

**(B) Second example of CDRH3 biased in vivo.**

As a second example, three groups of four mice were either kept naive or immunized with hIL6 receptor (153µg per mouse) or hIFNγ (92µg per mouse). Both targets were biotinylated and produced internally. CDRH3 were extracted from the animals’ spleens and captured as described before into the acceptor library of human frameworks. Resulting libraries, i.e. MnI (capturing naive CDRH3), MiJ (capturing CDRH3 from mice immunized with hIFNγ) and MiK (capturing CDRH3 from mice immunized with hIL6 receptor), were used in phage display selection against hIL6 receptor. Clones from the second selection round were then tested in phage screening ELISA according to the same procedure as described earlier.

**(C) Specificity ELISA.**

The three most amplified clones from each library were tested in dose response ELISA against a panel of irrelevant targets to ensure their specificity to hIFNγ. MnA-1 and 2 being very similar in sequence, MnA-1, 3 and 4 were chosen for testing. Streptavidin (Roche) was coated overnight at 4°C at 1µg/mL, 50µL per well on a maxisorb 96 wells plate (Nunc). Human IgG2 (VH4, Vκ6) (produced in house), hIgG3 (VH1,Vλ3) (produced in house), horse radish peroxidase (HRP) (Thermo scientific), vCCI-Fc (viral CC-chemokine inhibitor fused to a human Fc, produced in house), mTLR4-Fc (mouse toll like receptor four fused to a human Fc) (Alexis biochemicals) were coated overnight at 4°C at 2µg/mL, 50µL per well on a maxisorb 96 wells plate (Nunc). Plates were blocked with PBS containing 3% BSA for 1 hour. Biotinylated hen egg lysozyme (Sigma), and biotinylated hIgG1 (VH3, Vλ6) (produced in house) were coated for 30min at 2µg/mL in 50µL PBS BSA 1% on wells precoated with streptavidin. Biotinylated human IFNγ (produced in house) was coated similarly at 1µg/mL. Purified scFv were tested at 1100nM, 110nM and 11nM in 50µL BSA 1%. Most plates were revealed using and mouse anti-c myc and goat anti-human Fcγ HRP (Jackson) as described in the dose response ELISA section. For the plates coated with HRP, revelation was performed using an anti-mouse Fcγ coupled to alkaline phosphatase (Sigma) and the addition of 50µL 1-step PNPP substrate (Thermo scientific). After 30min incubation, plates were blocked with 50µL of NaOH (2M) and read was performed at 405nm. Controls of coating were performed with specific antibodies, i.e. a goat anti-human Fcγ HRP antibody (Jackson) for IgG1, 2, 3 and mTL4R-(hu)Fc, a rabbit anti-hen egg lysozyme HRP (Abcam), a mouse anti-HRP (Abcam), a mouse anti-vCCI (R&D) and an internal human IgG specific to hIFNγ. Streptavidin coating was checked via the coating of biot-IgG1 at 2µg/mL and detection with a goat anti-human Fcγ HRP antibody (Jackson). Control antibodies were tested on wells coated with the target and on wells without target to ensure their specificity.
